# Supplementary figures and images for: Hemoglobin alpha regulates T-lymphocyte activation and mitochondrial function
Source: Front Immunol. 2026 Jan 8;16:1725904. doi: 10.3389/fimmu.2025.1725904 (PMC12823933; doi:10.3389/fimmu.2025.1725904)

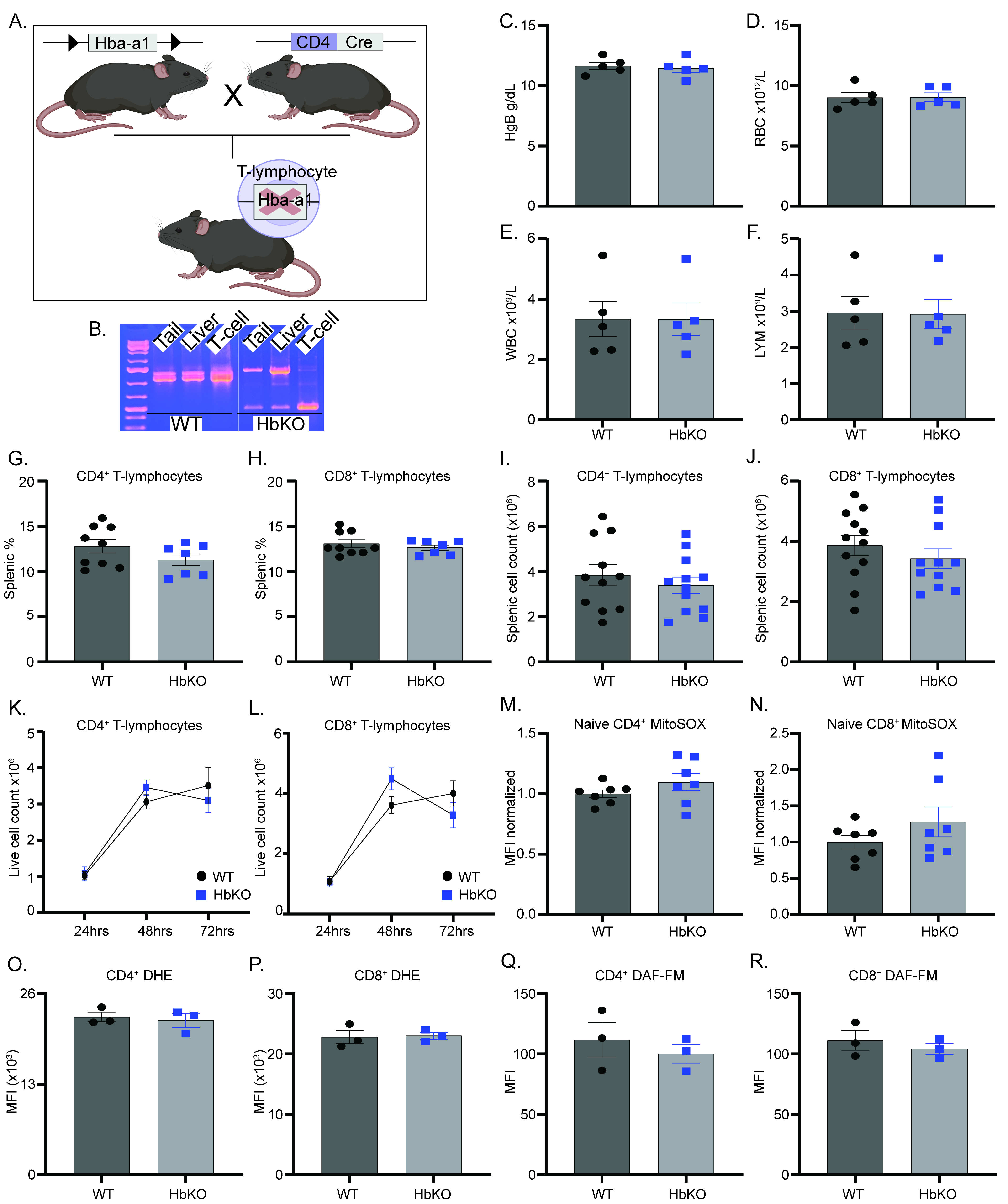

Supplement: Supplementary Figure 1 — Generation of T-lymphocyte specific hemoglobin alpha a1 knock-out animals. (A): Schematic of T-lymphocyte specific Hbα-a1 mouse knock out (HbKO) generation. (B): Results of tail, liver, and isolated pan T-lymphocytes genotyped for Hbα-a1 excision (WT: 1088bp, excised: ~500bp) (N = 1 per genotype). (C-F): Complete blood count results of hemoglobin (C), red blood cells (D), white blood cells (E), and lymphocytes (F) from whole blood samples from WT and HbKO animals (N = 5 per genotype). (G, H): Splenic percentages of CD4+(G) and CD8+(H) T-lymphocytes in WT and HbKO animals (WT N = 9, HbKO N = 7). I-J: Live cell counts of CD4+(I) (WT N = 11, HbKO N = 12) and CD8+ T-lymphocytes (J) (WT N = 12, HbKO N = 11) isolated from whole spleens. (K, L): Growth curves of activated isolated splenic CD4+(K) and CD8+(L) T-lymphocytes over 24, 48, 72 hours (N = 6 per genotype). (M-R): Naïve splenic CD4+ and CD8+ T-lymphocytes were isolated and measured for MitoSOX (M, N) (N = 7 per genotype), DHE (O, P) (N = 3 per genotype), and DAF-FM (Q, R) (N = 3 per genotype) (mean fluorescence intensity). Statistics (not significant, not shown) were measured using Student’s t-test or two-way ANOVA. [file Image1.tif]

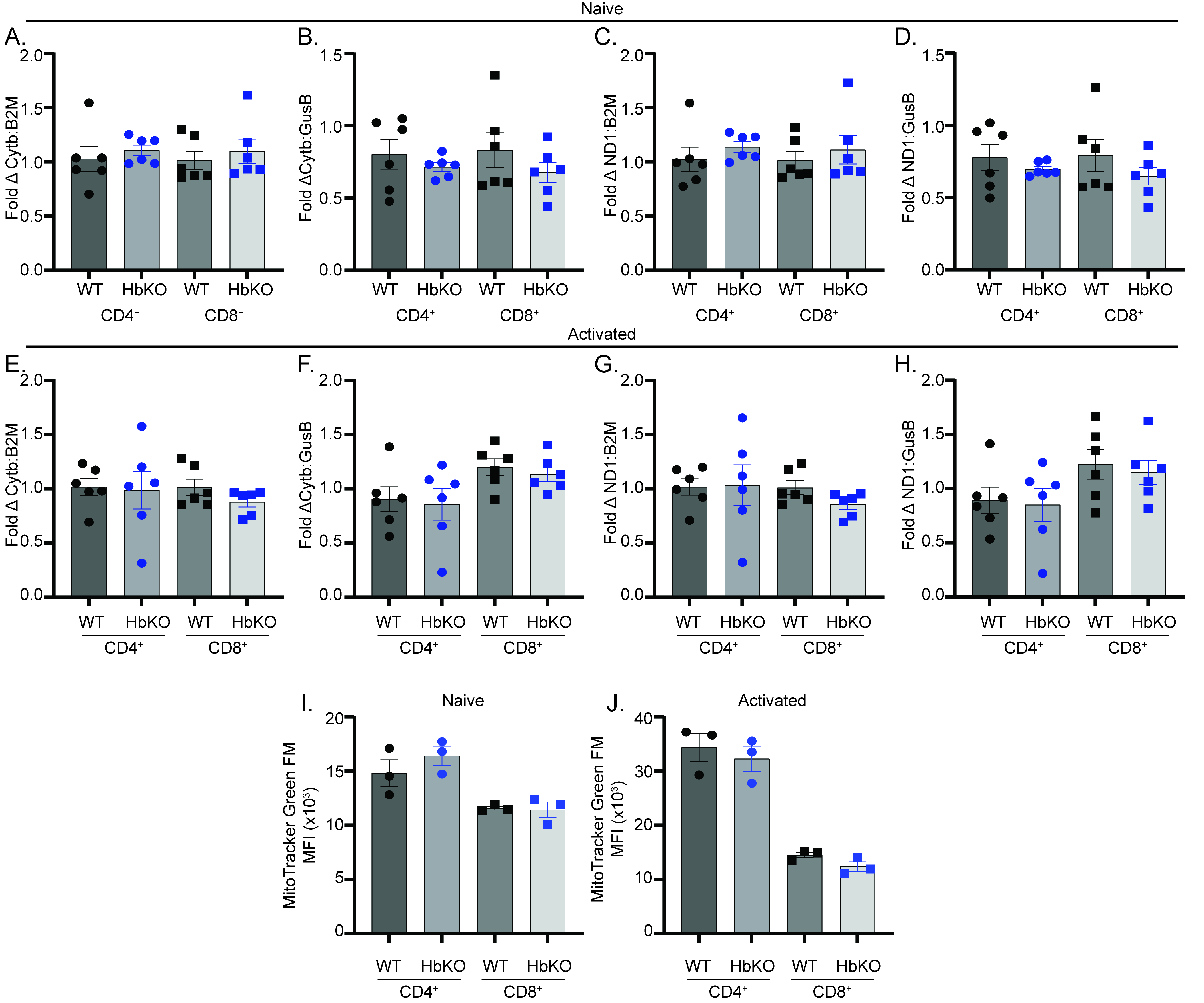

Supplement: Supplementary Figure 2 — Loss of Hba-a1 in T-lymphocytes does not affect mitochondrial number or mass. Naïve (A-D) and 72-hour activated (E-H) CD4+ and CD8+ T-lymphocytes from WT and HbKO animals were assessed for mitochondrial DNA content via qPCR (N = 6 per genotype). Mitochondrial mass was assessed in naïve (I) and 72 hour activated (J) T-lymphocytes by determining MFI of MitoTracker Green FM via flow cytometry (N = 3 per genotype). Statistics (not significant, not shown) were calculated using two-way ANOVA with Fisher’s LSD multiple comparisons. [file Image2.tif]

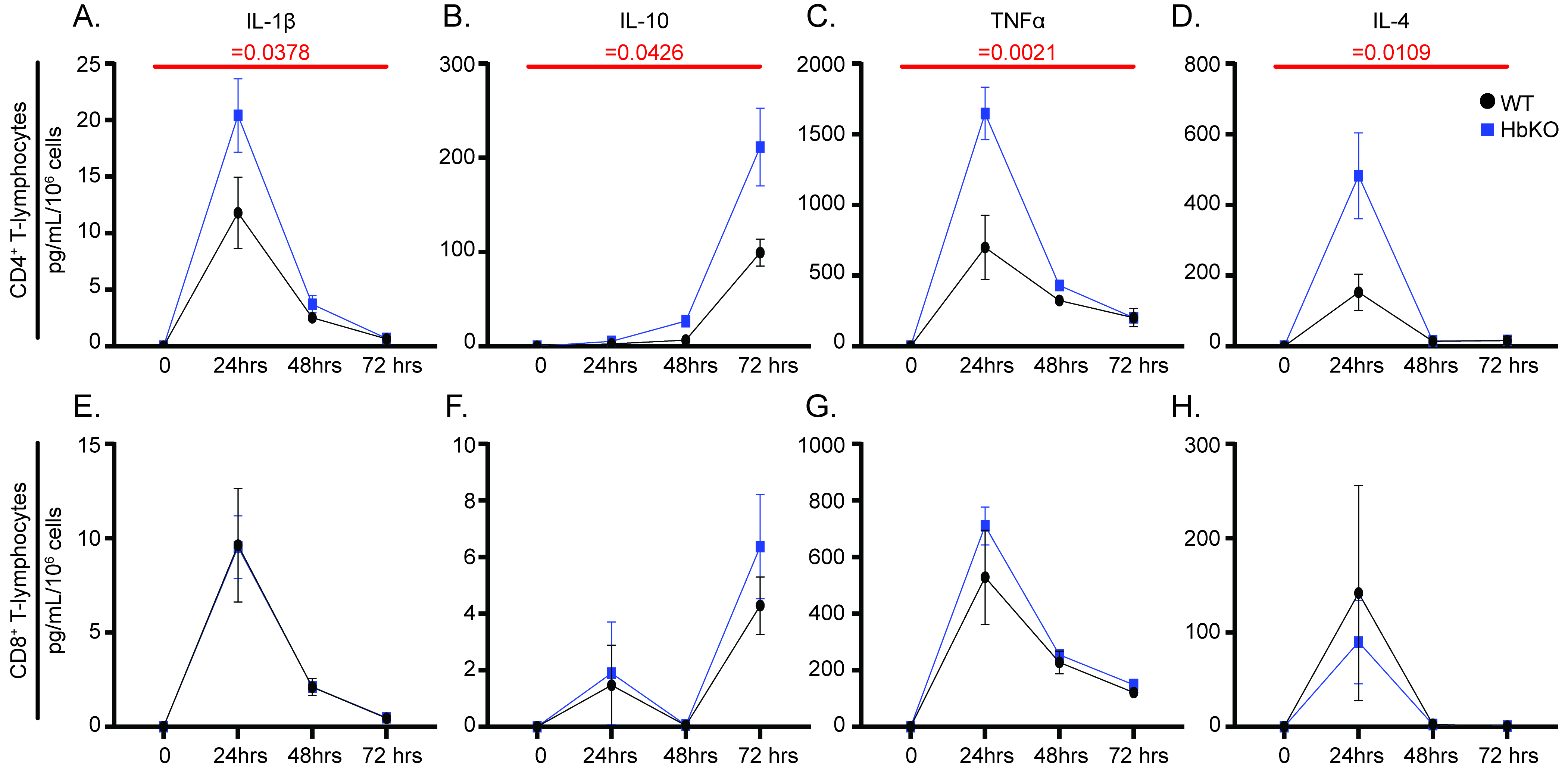

Supplement: Supplementary Figure 3 — Additional cytokines measured in T-lymphocyte time course activation. Extracellular cytokine protein concentration from activated CD4+(A-D) and CD8+ T-lymphocytes (E-H) from WT and HbKO animals (N = 5 per genotype). Statistics measured using two-way ANOVA. [file Image3.tif]

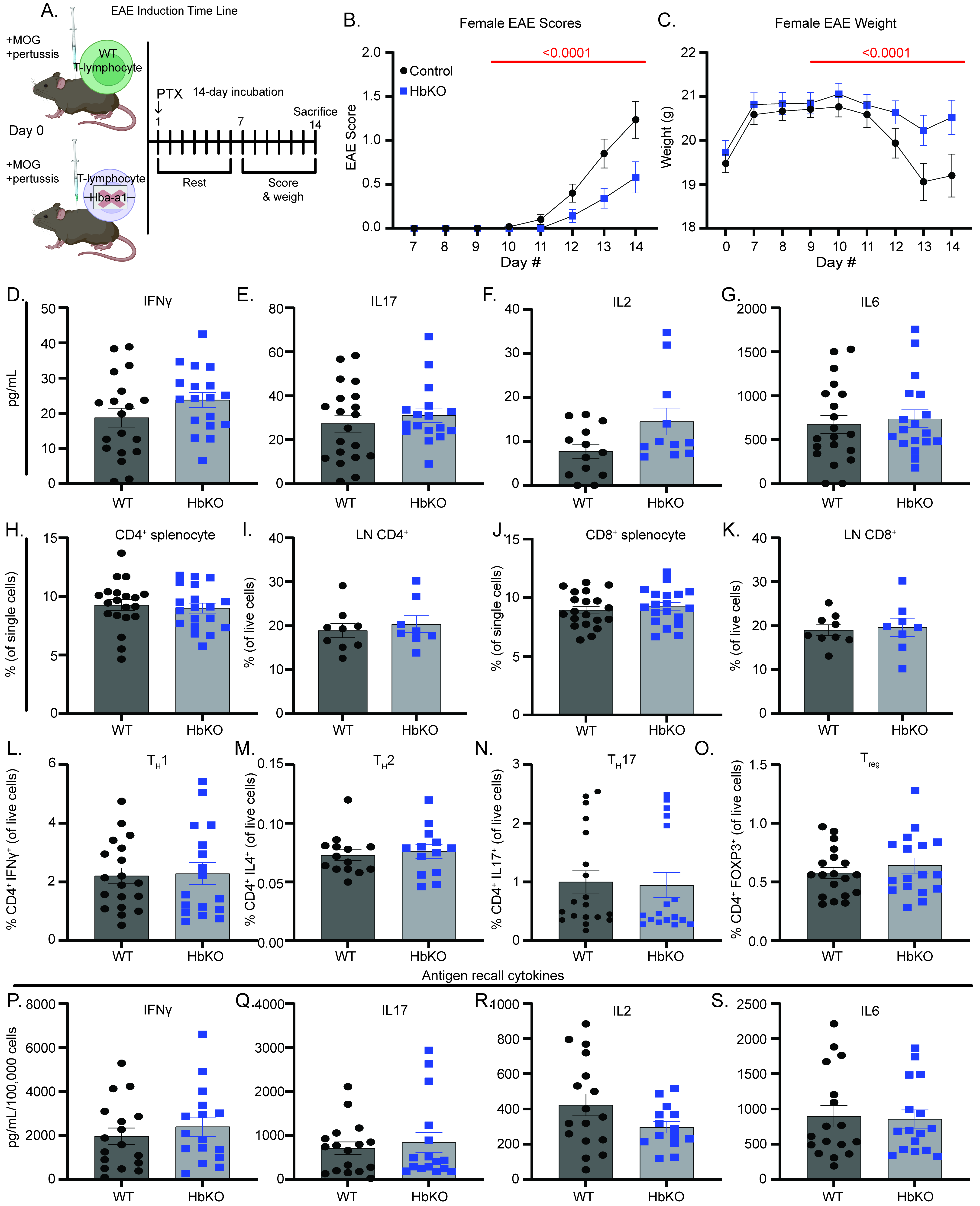

Supplement: Supplementary Figure 4 — Fourteen day EAE procedure. (A): Schematic of 14-day EAE experimental design. (B, C): EAE severity scores (0–5) and mouse weights (g) over 14-day incubation period (WT N = 30, HbKO N = 25). (D-G): Protein concentration (pg/mL) of cytokines in plasma. (H-K): Percentage of CD4+ and CD8+ present in the spleen (WT N = 20, HbKO N = 19) and lymph nodes (WT N = 9, HbKO N = 8)at day 14. (L-O): Percentage of splenic CD4+ polarized T-lymphocytes (WT N = 19, HbKO N = 17). (P-S): Splenocytes restimulated with 10 μg/mL MOG35–55 for 72 hours, then assessed for extracellular cytokine protein concentration (pg/mL per 106 cells) (WT N = 17, HbKO N = 16). Statistics measured by two-way ANOVA with Šídák’s multiple comparisons test or Student’s t-test where appropriate. [file Image4.tif]

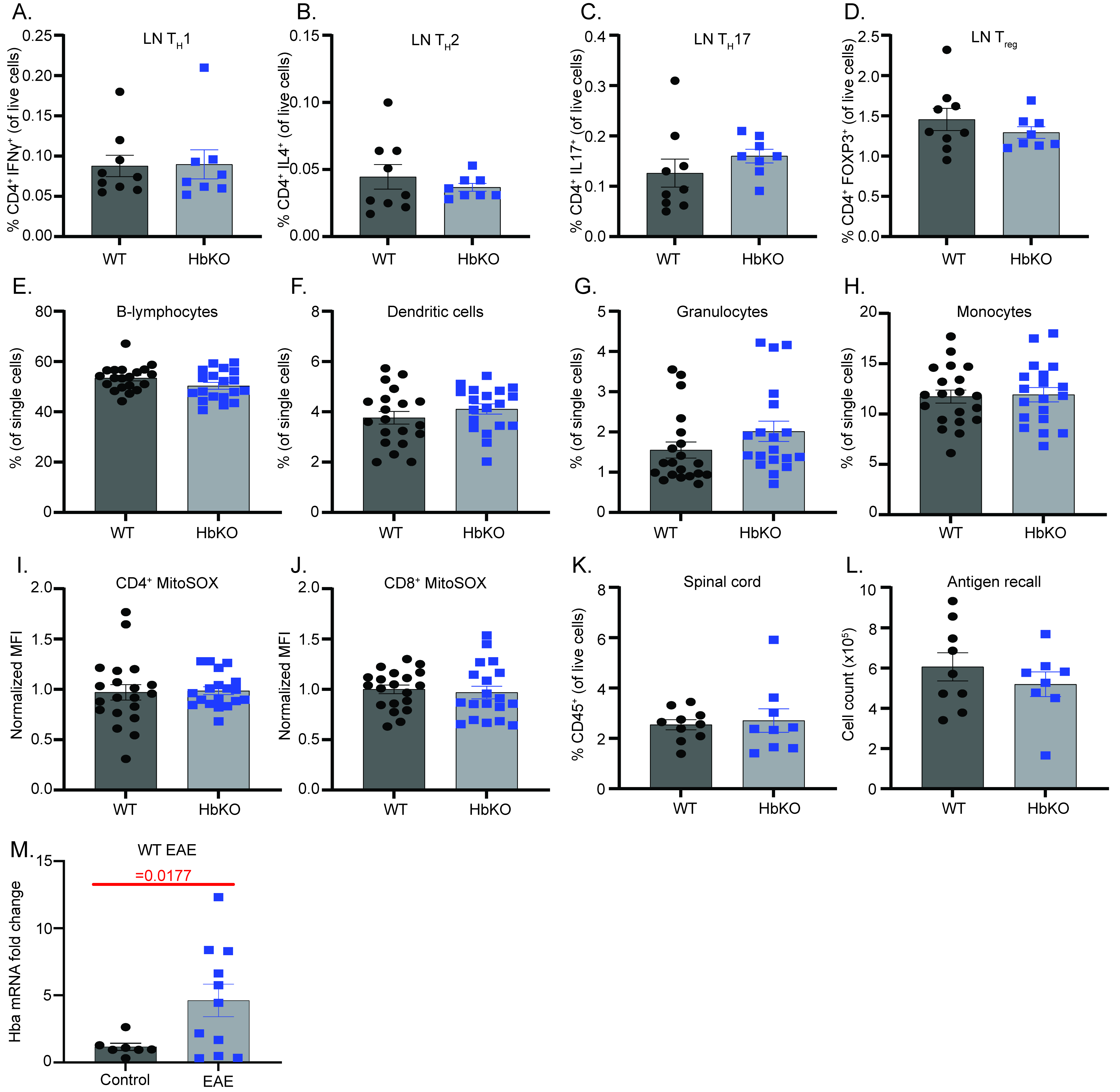

Supplement: Supplementary Figure 5 — Splenic and spinal cord immune cell percentages do not change between WT and HbKO EAE animals. (A-D): Percentage of inguinal lymph node CD4+ polarized T-lymphocytes. (E-H): Immune cell populations of B-lymphocytes (E), dendritic cells (F), granulocytes (G) and monocytes (H) were assessed by flow cytometry. (I, J): MitoSOX MFI of CD4+(I) and CD8+(J) splenic T-lymphocytes. (K): Percentage of CD45+ cells present in the spinal cord. (L): Live cell count of splenocytes after 72-hour restimulation with 10 µg/mL MOG35-55. (M): Splenic T-lymphocytes from control and 14 day EAE animals were assessed for Hba gene expression fold change. Statistics (not significant, not shown) were measured using Student’s t-test. [file Image5.tif]
